# *Enterococcus faecium* DNA in acute decompensated cirrhosis: a key player in inflammation and kidney dysfunction

**DOI:** 10.3389/fmed.2025.1629210

**Published:** 2025-10-20

**Authors:** Olaf Tyc, Nico Kraus, Toska Wiedemann, Hans-Peter Erasmus, Cristina Ortiz, Jessica Vasseur, Kai Hourfar, Christian Seidl, Marcus Maximilian Mücke, Holger Storf, Iulia Dahmer, Eva Herrmann, Stefan Zeuzem, Volkhard A. J. Kempf, Jonel Trebicka, Christoph Welsch, Angela Brieger

**Affiliations:** ^1^Medical Clinic 1, Biomedical Research Laboratory, Goethe University Frankfurt, University Hospital, Frankfurt am Main, Germany; ^2^Institute of Medical Informatics, Goethe University Frankfurt, University Hospital, Frankfurt am Main, Germany; ^3^Institute for Transfusion Medicine and Immunohematology, German Red Cross Blood Donation Service Baden-Wuerttemberg-Hessen, Frankfurt am Main, Germany; ^4^Institute of Biostatistics and Mathematical Modeling, Goethe University Frankfurt, University Hospital, Frankfurt am Main, Germany; ^5^Institute for Medical Microbiology and Infection Control, Goethe University Frankfurt, University Hospital, Frankfurt am Main, Germany; ^6^Department of Internal Medicine B, Faculty of Medicine, University of Münster, Münster, Germany

**Keywords:** bacterial translocation, *Enterococcus faecium*, acute-on-chronic-liver-failure, chronic liver diseases, inflammation, gut barrier, gut dysbiosis, microbiome

## Abstract

Liver cirrhosis is a major global health burden, with acute-on-chronic liver failure (ACLF) being a severe complication associated with high mortality. Systemic inflammation (SI) plays a crucial role in ACLF development, yet indicators for predicting disease progression remain limited. *Enterococcus faecium* (EF) has been implicated in bacterial translocation and SI, but its clinical relevance in ACLF remains unclear. We analyzed sera of 197 patients from a prospective observational study with acutely decompensated liver cirrhosis *versus* 234 healthy controls for the presence of EF DNA using RT-qPCR and cytokine analysis of serum samples. Overall, EF DNA was detected in 26% (*n* = 51, *p* = 0.001) of the patients, and only in 1.28% (*n* = 3, *p* = 0.001) in the control cohort. The positive patient samples were distributed as follows: 12% of patients were with stable decompensated cirrhosis (SDC), 5% of patients were with unstable decompensated cirrhosis (UDC) and 10% in patients were with ACLF. In the latter group, EF positivity significantly correlated with significant elevated leukocyte counts, increased C-reactive protein (CRP), Interleukin-6, and increased bilirubin, Aspartate Aminotransferase (AST), as well as creatinine levels. These findings suggest that the translocation of EF or its DNA, into the systemic circulation may reflect increased intestinal permeability, which is thought to be a key driver of SI and subsequent organ failure in ACLF. Taken together, our findings demonstrate that the presence of EF DNA in serum may contribute to the pathophysiological cascade of ACLF by promoting SI and organ dysfunction, particularly affecting renal function. We therefore propose and hypothesize that the presence of EF DNA in patients’ serum could serve as an indicator of intestinal barrier dysfunction and further underscores the critical role of the gut-liver axis in the development and progression of ACLF.

## Introduction

Cirrhosis is the common end stage of most chronic liver diseases. Complications of cirrhosis are responsible for over 2.0 million deaths worldwide every year and rank as the 11th most common cause of death globally, and the ninth leading cause in Central Europe (1). The transition from compensated to decompensated liver cirrhosis is a hallmark of disease progression, however, predictors to assess the risk of decompensation in individual patients from routine diagnostics are lacking. Depending on the grade of decompensation, the one-year mortality rate varies between 1 and 57% over the course of disease. A dreaded course of acute decompensation is acute-on-chronic liver failure (ACLF), a fatal disease with rapid deterioration, with no effective treatment options available. Half of the patients with acute decompensation from liver cirrhosis develop ACLF, and ∼40% of these die within 28 days (2–7). The pathogenesis of ACLF is poorly understood. Various precipitating events are believed to induce dysfunction or failure of the liver and other organs. The PREDICT study uncovered three clinical courses of acutely decompensated liver cirrhosis that showed distinct pathophysiology (8). First pre-ACLF patients did not initially meet CLIF ACLF criteria at decompensation but developed ACLF within 3 months, with high 3-month and 1-year mortality. Second, patients with unstable decompensated cirrhosis (UDC) required ≥1 readmission. Third, stable decompensated cirrhosis (SDC) patients did not develop ACLF and had much lower mortality. Beyond portal hypertension, the grade and trajectory of systemic inflammation (SI) distinguished the groups Pre-ACLF and ACLF patients had high-grade and worsening SI, while UDC and SDC patients showed low-grade SI—steady in UDC, improving in SDC. The PREDICT study emphasized the need to identify factors driving SI exacerbation and biomarkers to predict progression after acute decompensation (4, 8). Bacterial infections are the most prevalent triggering factor for ACLF in the western world, occurring in up to 37% of cases and are more commonly observed as a precipitating event in patients with ACLF compared to those without ACLF (3, 6, 9). The bacterial infections most frequently associated with ACLF triggers include spontaneous bacterial peritonitis (SBP), pneumonia, and urinary tract infections (UTI). In many cases, these infections are caused by Gram-positive bacteria, such as *Staphylococcus aureus*, *Enterococcus faecalis*, and *Enterococcus faecium* (EF), as well by Gram-negative bacteria, including *Escherichia coli*, *Klebsiella pneumoniae*, and other *Enterobacteriaceae* (3, 9, 10).

One of the key exogenous factors implicated in the development of ACLF in individuals with liver diseases is bacterial translocation. This process involves the passage of bacterial components, such as pathogen-associated molecular patterns (PAMPs) and bacterial metabolites, across a compromised intestinal barrier. The disruption of the epithelial barrier enables bacterial components to enter the bloodstream, potentially triggering SI and exacerbating liver dysfunction (3, 10–14). Recent gut microbiome studies have shown that bacteria of the genus *Enterococcus* including EF and other oral bacterial species such as *Streptococcus oralis* were significantly enriched in the gut of patients with liver cirrhosis (10, 15). The increased abundance of *Enterococcus* spp. has been linked to an elevated Model for End-Stage Liver Disease (MELD) score and higher Child-Pugh scores, as well to organ failure in affected patients (15). Additionally, *Enterococcus* spp. have been found to be more abundant in patients with liver cirrhosis who have died (15). The presence of PAMPs and bacterial metabolites, particularly from EF, may act as triggers for kidney dysfunction and failure. Excessive SI in ACLF patients can result in the activation and dysfunction of the innate immune system, which is challenged by increased PAMPs and damage-associated molecular patterns (DAMPs) (16, 17). SI leads to cell and tissue immunopathology, contributing to hepatic and extrahepatic organ failure, including the kidneys (17, 18). In addition to the effects of PAMPs, several cytokines can mediate dysfunction of the intestinal barrier (19, 20): TNFα, IFNγ, and interleukin (IL)-1β are among the most extensively studied cytokines that promote increased intestinal permeability (20–22). Understanding the role of SI and the impact of specific bacterial species, such as EF, on organ dysfunction particularly kidney dysfunction is crucial for developing targeted therapies to prevent or mitigate organ failure in progressive liver diseases and ACLF.

The aim of the present study was to investigate the prognostic significance of the detection of EF DNA in the serum of patients with acute decompensation of liver cirrhosis, with a particular focus on its association with systemic inflammation and organ dysfunction, including the development of ACLF. To this end, serum samples from 197 patients were analyzed as part of a prospective cohort study on acute decompensation and ACLF. Clinical markers of systemic inflammation, organ failure, and relevant cytokines were characterized and correlated with EF DNA detection.

## Materials and methods

### Patients

A total of 197 patients that entered the prospective ACLF-I cohort study (observational study for the characterization of the pathogenesis of ACLF) between November 2020 and April 2023 and were included in this study (7). Patients with decompensated liver cirrhosis, age between 18 and 80 years, were eligible to enter the study. Hepatocellular carcinoma outside MILAN criteria, other malignancy, or severe congenital/acquired immune deficiency (e.g., HIV, immunosuppressive therapy in transplant recipients or rheumatologically diseases) and pregnancy were exclusion criteria. Demographic, laboratory and clinical characteristics are systematically recorded in a digital patient register (OSSE) (23, 24) from the clinical care data in cooperation with the Institute for Medical Informatics and the Data Integration Center at the Goethe University Hospital, Frankfurt (DIZ). Serum samples were collected on the day of study inclusion (baseline) and during follow-up. ALCF was classified according to the CLIF score and the European Association for the Study of the Liver (EASL)-CLIF criteria (17, 25). Alcohol-related liver cirrhosis was defined by a reported daily drinking average above 20 g/dL in their patient history. A cohort of healthy individuals (*n* = 234) was added as a control group. Of these, 200 samples were provided by the DRK Blood Donation Service Baden-Württemberg/Hessen, Frankfurt am Main, Germany, and 34 samples were obtained from healthy volunteers at Frankfurt University Hospital. Prior to participation, all participants gave their written informed consent.

### Ethics approval

This study was performed in accordance with the declaration of Helsinki and approved by the local ethics committee (ethics vote no. 20–653). All patients gave their written informed consent prior to entering the study.

### Blood sampling and data collection

Clinical data, laboratory data and serum samples were obtained at baseline and follow-up (obtained at each visit during a 3-month follow-up). Routine laboratory diagnostics include liver function tests differential white blood cell count (WBC), C-reactive protein (CRP), drinking behavior, smoking behavior, gastrointestinal bleeding, ascites, therapeutic paracentesis, albumin treatments, diabetes and diabetes treatment, transjugular intrahepatic portosystemic shunting (TIPS), hepatitis, viral infections, renal failure, respiratory failure, circulatory failure, data on bacterial and/or fungal infection development. Laboratory data and biological samples were obtained at each visit during a 3 month follow up.

### Processing of blood samples and serum isolation

Serum samples (9 mL) were taken from patients and healthy individuals and subjected to centrifugation. Serum samples were centrifuged at 1.400 x g for 10 min at 4 °C and the supernatant was taken, aliquoted and stored at −80 °C until further use.

### Bacterial DNA isolation and quantification

Genomic DNA was isolated from 250 μL serum using the QIAmp® DNA Blood Mini Kit (Qiagen, Germany, Cat# 51104) according to the manufacturer’s manual under sterile conditions within a laminar flow cabinet. Prior to each extraction, the flow cabinet was decontaminated using a two-step disinfection procedure: initially with 2% Incidin Plus (Ecolab), which was applied for 15 min. to eliminate a broad spectrum of microorganisms, followed by 80% ethanol to remove residual disinfectant and enhance surface sterility. Subsequently, the cabinet was exposed to ultraviolet (UV) light for 30 min. to ensure additional decontamination under sterile conditions. To monitor for potential bacterial DNA contamination, two negative extraction controls (columns processed without serum) were included in the DNA extraction steps. The isolated DNA was eluted in 35 μL Elution Buffer (EB) and DNA concentration and purity were assessed using a NanoDrop® ND-2000 spectrophotometer (Thermo Fisher Scientific).

### Bacterial strains and culture conditions

The following bacterial isolates were used in this study: *E. faecium* (DSM# 20477), *E. coli* Dh5α and *E. coli* JM109 (Promega Corp. Madison, US cat# L2005). The bacterial strains were pre-cultured from −80 °C glycerol stocks on either lysogeny broth (LB)- agar plates (LB-Agar Lennox, Carl Roth GmbH + Co. KG, 37 gL^−1^) or on Trypticase soy yeast extract medium (TSYM) agar plates (30 gL^−1^ Carl Roth Trypticase soy broth, 2.0 gL^−1^ Carl Roth Yeast Extract, 15 gL^−1^ Sigma-Aldrich Agar). The bacteria were incubated at 37 °C on agar plate’s prior application. All bacteria used in this study are listed in Supplementary Table S1.

### Cloning of 16S rDNA gene fragments in pGEM-t-easy vector for real time-quantitative PCR standards

For the quantification of EF 16S rDNA, a sequence fragment of the 16S rDNA of EF were cloned into the pGEM-T vector (26, 27) and the corresponding plasmids were used to establish real-time-quantitative PCR (RT-qPCR) standard curves. Briefly, a standard PCR was performed using a 50 μL PCR- GoTaq™ green master mix (Promega Corp. Madison, US cat# M712) containing 1 μL of EF DNA. For the 16S rDNA gene amplification EF specific primers were used: forward primer *E. faecium*_qPCR_16SF (5′-GCGGCTCTCTGGTCTGTAAC-3′), reverse primer *E. faecium_*qPCR_16SR (5′- TAAGGTTCTTCGCGTTGCTT-3′), amplifying ∼254 bp from the 16S rDNA gene of EF. The amplified PCR products were checked on 1.5% agarose gel and the PCR products were cut out and purified with the QIAquick® Gel Extraction Kit (Qiagen, Cat# 28704). The purified PCR products were ligated into the pGEM-T vector (Promega, Madison, WI, US cat#A137A) using Promega T4 DNA Ligase (Promega Corp. Madison, US cat# M180A) and transformed into *E. coli* JM109 competent cells (Promega, Madison, WI, US cat# A1380) according to the manufacturer’s manual instructions. Clones were picked and checked for the correct insert by using the M13 forward and reverse primers and sequencing of the PCR products by Sanger-Sequencing (EUROFINS, Ebersberg, Germany).

### Quantification of EF in serum samples via RT-qPCR

The abundance of EF in serum samples was measured by RT-qPCR using the above mentioned primers: *E. faecium*_qPCR_16SF, reverse primer *E. faecium*_qPCR_16SR, amplifying ∼254 bp from the 16S rDNA gene of EF From each sample 2 μL DNA were subjected to quantitative RT-qPCR using QuantiNova® SYBR® Green PCR master mix (Qiagen, Germany, Cat# 208054). For quantification two-step quantitative RT-qPCRs was performed on an Applied Biosystems StepOnePlus Real-Time PCR System (Applied BioSystems, Waltham MA, United States), with the following settings: initial cycle 95 °C for 2 min., followed by 40 cycles of 95 °C for 5 s. (denaturation) and 60 °C for 10 s. (combined annealing/extension). All samples were run in triplicate. Negative controls included nuclease-free water (no-template control, NTC) and eluted DNA from the negative extraction controls (columns processed without serum; NC). These controls were included in each RT-qPCR reaction to monitor for contamination (28, 29). To avoid cross-contamination, RT-qPCR reactions were prepared in a separate room and under a different laminar flow hood than those used for DNA extraction. RT-qPCR efficiency was evaluated using a standard curve generated from fivefold serial dilutions (in triplicate) of plasmid DNA (pGEM-T vector) containing the respective *E. faecium* 16S rDNA gene fragment, or genomic DNA from *E. faecium*. The calculated amplification efficiencies ranged from 87 to 107%, with correlation coefficients (R^2^) between 0.91 and 0.98. Relative gene expression was quantified using the 2^−ΔΔ Ct method, normalized to the 16S rDNA gene expression levels of the standards (30). All primers used in this study are listed in Supplementary Table S2.

### 16S rDNA gene sequencing of positive serum DNA samples

For RT-qPCR samples that were tested positive for EF DNA, the PCR products were extracted and purified using the QIAquick® PCR Purification Kit (Qiagen, Germany, Cat# 28104), following the manufacturer’s instructions. For molecular identification through Sanger sequencing, 15 μL of the purified PCR product DNA was premixed with 2 μL of primer (final concentration 10 pmol/μL). For 16S rDNA gene sequencing the following primer was used: E.faecium_qPCR_16SF (5’-GCGGCTCTCTGGTCTGTAAC-3′) amplifying ∼254 bp from the 16S rDNA gene of EF. The premixed sequencing samples were sent to Eurofins for Sanger-sequencing using the TubeSeq service (Eurofins Genomics Europe, Ebersberg, Germany).

### Quantification of cytokine levels in serum samples with Luminex® human discovery assays

To determine the concentration of inflammatory cytokines, present in the serum samples we measured the cytokine concentration of 11 different cytokines IFN-gamma (BR29), IL-1 alpha/IL-1F1 (BR38), IL-1 beta/IL-1F2 (BR28), IL-1ra/IL-1F3 (BR30), IL-2 (BR43), IL-6 (BR13), IL-10 (BR22), IL-18/IL-1F4 (BR78), Lymphotoxin-alpha/TNF-beta (BR45), MIF (BR53), TNF-alpha (BR12) in all serum samples of the patients by using Luminex® Discovery Assay (Bio-Techne GmbH Cat# LXSAHM-16). The cytokine measurements were performed on a BioPlex 200 system powered by Luminex® xMAP™ Technology and xPONENT software V4.3 according to the manufacturer’s manual.

### Correlation analysis and random forest analysis

Prior to correlation analysis and random forest analysis, data normalization and scaling were performed using log transformation (base 10) and auto scaling (Z-transformation of each variable). Kendall or Spearman rank correlation tests were conducted to evaluate the features of interest. To determine the significance of each clinical variable, its contribution to the clinical phenotype, and its association with the presence of EF DNA, Random Forest analyses were performed using 500 trees for supervised classification. These analyses were carried out with the R software and the MetaboAnalyst package (31–33). The mean decrease in accuracy was calculated as a measure of each variable’s importance and subsequently plotted.

### Statistical analysis and modelling of clinical data

All variables were plotted as single data point or expressed as median (interquartile range) and were compared between the EF positive (EF+) and EF negative (EF-) groups, organized by the respective stratification classes (SDC, UDC and ACLF). In violin plots quartiles are denoted as dotted black lines, medians are denoted as joined lines. Prior to statistical analysis, a normality test was conducted. Normally distributed data were analyzed using a t-test for univariate analysis. Influence of EF and disease stages (SCD, UCD and ACLF) on different endpoints was investigated with the full model two-way ANOVA followed by a post-hoc Tukey test (HSD-test). For non-normally distributed data the Kruskal-Wallis test was used. Chi-square tests or Fisher’s exact test was performed to compare aetiologies and types of acute decompensation as well as to compare the frequency of EF in patients and controls. *p*-values ≤ 0.05 were considered to be statistically significant. Statistical calculations and plots of cytokine intensities and clinical lab values for all individual patients were performed and created using GraphPad Prism version 9.5.1 for Windows (GraphPad Software, San Diego, California, United States).^1^

## Results

### Patients’ characteristics

**Figure 1:**
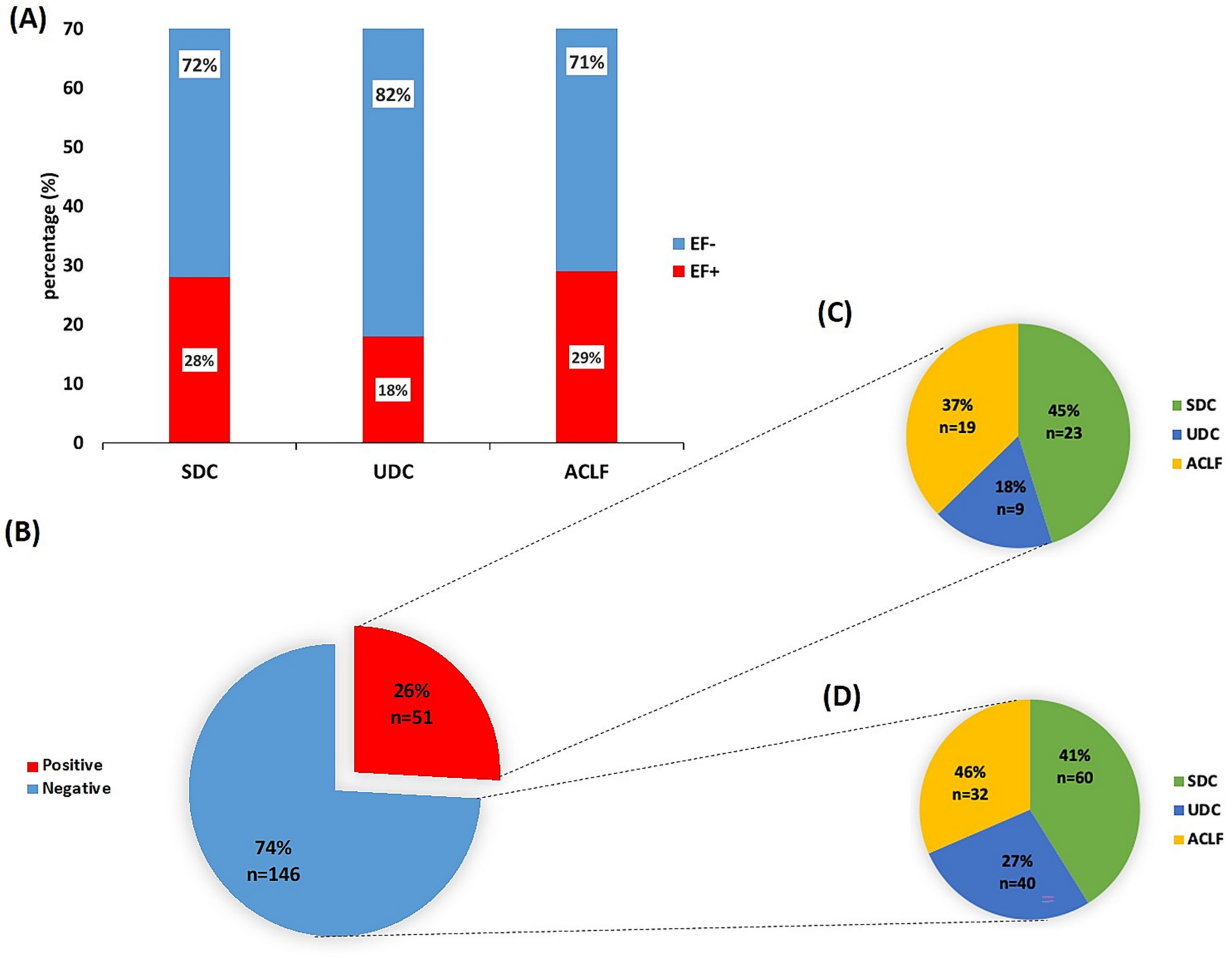
Detection of *E. faecium* DNA in sera from patients with unstable/stable decompensated cirrhosis or ACLF. **(A)** Overview over the patient cohort (*n* = 197) stratified according to the PREDICT study guidelines into stable decompensated cirrhosis (SDC), unstable decompensated cirrhosis (UDC) and acute-on-chronic liver failure (ACLF) and presence/absence of *E. faecium* (EF) DNA. The cohort included 83 patients with SDC, 49 with UDC and 65 patients with ACLF. **(B)** Real time -quantitative PCR (RT-qPCR) results of serum samples from the patient cohort classified into EF DNA-positive (EF+) and EF DNA-negative (EF-) groups. **(C)** EF DNA-positive patients and **(D)** EF DNA negative patients characterized (in %) across the different disease classes: SDC, UDC and ACLF.

In total, blood samples of 197 patients who were prospectively enrolled in the above mentioned observational cohort were analyzed. Of these, 135 patients were male (69%) and 62 females (31%), average age 59 years (± 12). A control cohort, totaling 234 healthy individuals, consisted of 98 men (42%) and 136 women (58%), average age 41 years (± 16). Demographic and baseline characteristics of the patient cohort are depicted in Table 1. The classification of the patients’ clinical course (based on the PREDICT study criteria) identified 83 patients (42%) with SDC, of whom 28% were EF+ and 72% were EF-. Additionally, 49 patients (25%) were categorized with unstable decompensated cirrhosis (UDC), with 18% EF+ and 82% EF-. Furthermore, 65 patients (33%) developed acute-on-chronic liver failure (ACLF) within 3 months of hospital admission, with 29% EF+ and 71% EF- (Figure 1A). The prevalence of bacterial infections at baseline did not differ significantly between EF+ and EF- patients.

**Table 1:** Baseline characteristics of patients at study enrollment.

Diseases phenotype	SDC	UDC	ACLF
Patients total *n* = 197	*n* = 83 (42%)	*n* = 49 (25%)	*n* = 65 (33%)
Age, years, mean ±SD years	57.66 ± 13.12	62.17 ± 11.57	56.43 ± 11.8
Male (n, %)	61 (73%)	32 (65%)	42 (65%)
Female (n, %)	22 (27%)	17 (35%)	23 (35%)
*Enterococcus faecium* DNA + (n, %)	23 (28%)	9 (18%)	19 (29%)
*Enterococcus faecium* DNA (n, %)	60 (72%)	40 (82%)	46 (71%)
ACLF grades			
pre-ACLF	-	-	23 (35%)
ACLF-1	-	-	17 (26%)
ACLF-2	-	-	14 (22%)
ACLF-3	-	-	11 (17%)
Laboratory Data			
Sodium (mmol/L)	136.46 (119–149)	136.14 (123–144)	136.51 (125–157)
Serum creatinine (mg/dl)	1.08 (0.36–3.65)	1.22 (0.33–4.99)	1.95 (0.44–6.75)
Bilirubin (mg/dl)	0.2 (0.2–31.3)	3.09 (0.7–22.0)	14.54 (0.4–40.7)
ALT (Units/L)	45.39 (8–279)	44.45 (7–279)	37.19 (11–279)
AST (Units/L)	97.31 (20–502)	74.38 (12–318)	69.38 (20–318)
y-GT (Units/L)	146.23 (13–918)	114.23 (8–556)	96.89 (8–891)
ALP (Units/L)	191.92 (46–806)	173.26 (46–806)	172.36 (64–806)
CRP (mL/L)	2.83 (0.04–12.97)	3.41 (0.13–18.06)	2.67 (0.2–8.95)
Albumin (g/L)	3.15 (1.7–4.5)	3.03(1.8–4.30)	3.09 (1.8–4.3)
INR	1.43 (0.9–3.43)	1.53 (0.98–2.81)	2.05 (0.96–8.61)
Leucos (cells/L)	6.91 (1.09–17.04)	6.27 (0.69–19.46)	11.29 (3.04–26.25)

### Analysis of serum samples reveal 26% positivity for EF DNA by RT-qPCR

From the patients with acute decompensation of liver cirrhosis, sera of 51 patients (∼26%) were positive for EF DNA (EF+; by RT-qPCR), while sera of 146 patients (74%) were negative for EF DNA (EF-; Figure 1B). In contrast, only 3 samples (1.3%) from the control cohort were EF+ (*p*-value = 0.001; Supplementary Figure 1). In the three EF+ cases from the control cohort, the frequency of EF DNA reached up to 1.93 × 10^4^ copies of the 16S rDNA gene, corresponding to a theoretically estimated abundance of approximately 3.2 × 10^3^ EF CFUs, assuming 6 copies of the 16S rDNA gene per EF cell (34). In the patient cohort, EF DNA was detected at abundances of up to 3.5 × 10^5^ copies of the 16S rDNA gene, corresponding to approximately 5.8 × 10^4^ EF CFUs, based on an estimated six copies of the 16S rDNA gene per EF bacterial cell (34). Among EF+ patients, 23 (45%) were classified with having SDC, 9 (18%) classified with UDC, and 19 (37%) developed ACLF (Figure 1C). In the EF- group, 60 patients (41%) had SDC, 40 (27%) had UDC, and 32 (46%) were categorized with ACLF (Figure 1D). Of the 65 ACLF patients, 19 (29%) were EF+, with no significant differences in ACLF grades 1 to 3 between EF+ and EF- patients (data not shown). RT-qPCR analysis revealed significantly lower CT values in ACLF patients (mean CT=35.80) compared to those with SDC (mean CT=37.48, *p*< 0.0001) and UDC (mean CT=36.81, *p*= 0.0254; Supplementary Figure 2), indicating a higher abundance of EF 16S rDNA in their serum samples.

### Comparison of classical microbiological diagnostics and RT-qPCR detection of EF DNA

To assess the concordance between conventional microbiological diagnostics and molecular detection of EF DNA, we compared results from standard clinical specimens—blood cultures, urine samples, and VRE swab tests—with serum-based RT-qPCR results obtained from the same patient cohort (n=51). Classical microbiological analyses identified EF in a total of 7 patients: 1 in blood culture, 5 in urine samples, and 7 in VRE swabs (note that some patients had multiple positive specimen types). In contrast, RT-qPCR targeting the 16S rDNA of EF detected bacterial DNA in 51 out of 51 patients, indicating a substantially higher sensitivity. A Chi-square test confirmed that this difference is highly statistically significant (χ^2^ = 64.68, *p*< 0.0001), supporting the conclusion that RT-qPCR is markedly more sensitive than classic microbiological-based diagnostic methods in this cohort (Supplementary Figure 3).

### Comparison of aetiologies and portal hypertension

A chi-square analysis revealed a significant difference in the distribution of liver disease aetiologies between EF+ and EF- patients with ACLF (*p*= 0.0008), indicating a potential link between the underlying cause of liver disease and the detection of EF DNA in serum (Supplementary Figure 4A). Additionally, there was a significantly (*p*= 0.0254) higher proportion of EF+ patients with ACLF presented with portal hypertension compared to EF– patients. This finding suggests a potential association between the presence of *Enterococcus faecium* DNA and advanced portal hypertension in ACLF (Supplementary Figure 4B).

### EF DNA positivity correlates with elevated leukocyte counts, CRP and IL-6 indicating enhanced inflammatory response

**Figure 2:**
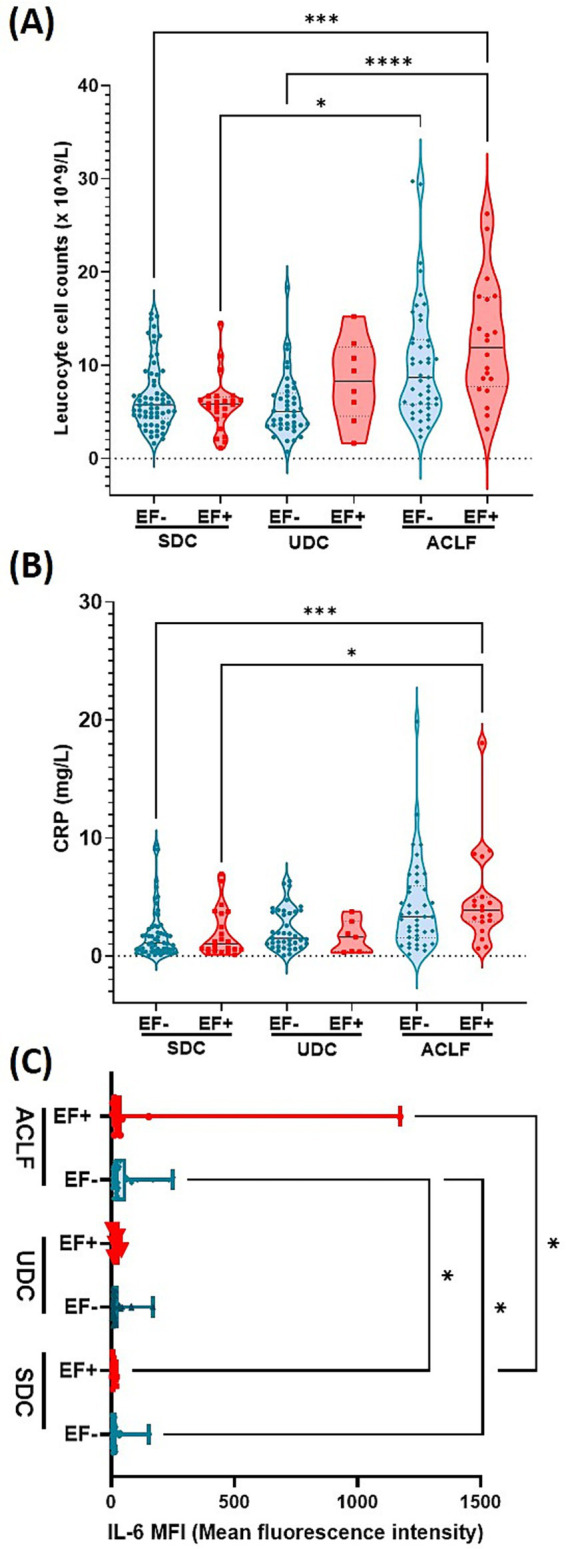
*E. faecium* DNA positivity is associated with elevated leukocyte count, CRP, and IL-6, indicating intensified inflammation in advanced cirrhosis stages. Plasma levels of Leucocyte count **(A)**, CRP **(B)** and IL-6 **(C)**. Significant differences were observed between patients who were *E. faecium* (EF) DNA negative (EF-) and those who were EF DNA positive (EF+), across the following patient groups: stable decompensated cirrhosis (SDC), unstable decompensated cirrhosis (UDC) and acute-on-chronic liver failure (ACLF), indicated by asterisk (*p< 0.05, ***p< 0.005, ****p< 0.0001). *p*-values were determined using one-way ANOVA with Kruskal-Wallis test or with FDR and Benjamin Hochberg correction (Figure 2c). Color codes: turquoise = EF-; red = EF+.

The analysis of inflammation-related markers between EF+ and EF- patients stratified by disease phenotype revealed significant differences in leukocyte counts, CRP, and IL-6 levels (Figure 2). Leukocyte counts were the highest in ACLF EF+ patients, followed by ACLF EF- patients. Both ACLF groups exhibited significantly higher leukocyte levels compared to UDC and SDC patients (*p*≤ 0.0001 and *p*≤ 0.005, respectively; Figure 2A). Among UDC patients, EF+ individuals tend to show elevated leukocyte counts relative to EF- patients, with significant enhanced leucocytes in ACLF EF+ compared UDC EF- patients (*p*≤ 0.0001; Figure 2A). In contrast, SDC patients had the lower leukocyte counts and showed no significant differences between the SDC EF+ and EF- group but a significant difference could be observed between SDC EF- and ACLF EF+ (*p*≤ 0.005) as well as SDC EF+ and ACLF EF- (*p*≤ 0.05; Figure 2A). These results underscore a possible link between EF DNA positivity and SI, particularly in patients with advanced disease stages such as ACLF and UDC. C-reactive protein (CRP) levels (Figure 2B) followed a similar trend. ACLF EF+ patients had the highest median CRP concentrations compared to EF+ and EF- SDC patients (*p*< 0.005; Figure 2B). Among UDC patients, there was no significant difference between EF+ and EF- individuals (*p*≥ 0.05). In the SDC group, CRP levels remained low overall, with no significant differences between EF+ and EF- patients (Figure 2B). As shown in Figure 2C, IL-6 levels were markedly elevated in ACLF EF+ patients, who exhibited the highest median concentrations among all liver disease phenotypes IL-6 levels in ACLF EF+ patients were significantly higher than those in SDC EF+ patients (*p*≤ 0.05). Additionally, IL-6 levels were significantly elevated in ACLF EF− patients compared to both SDC EF+ and SDC EF− patients (*p* < 0.05; Figure 2C). Among all groups, SDC EF+ patients had the lowest IL-6 levels, with no significant differences between EF+ and EF- individuals in the SDC category (Figure 2C). These observations point to a potential link between IL-6 elevation and EF DNA positivity in more advanced stages of liver disease. Overall, the data support the idea that EF DNA may contribute to an intensified SI response, particularly in UDC and ACLF patients.

### Liver dysfunction markers increase with disease severity and are the highest in EF+ ACLF patients

**Figure 3:**
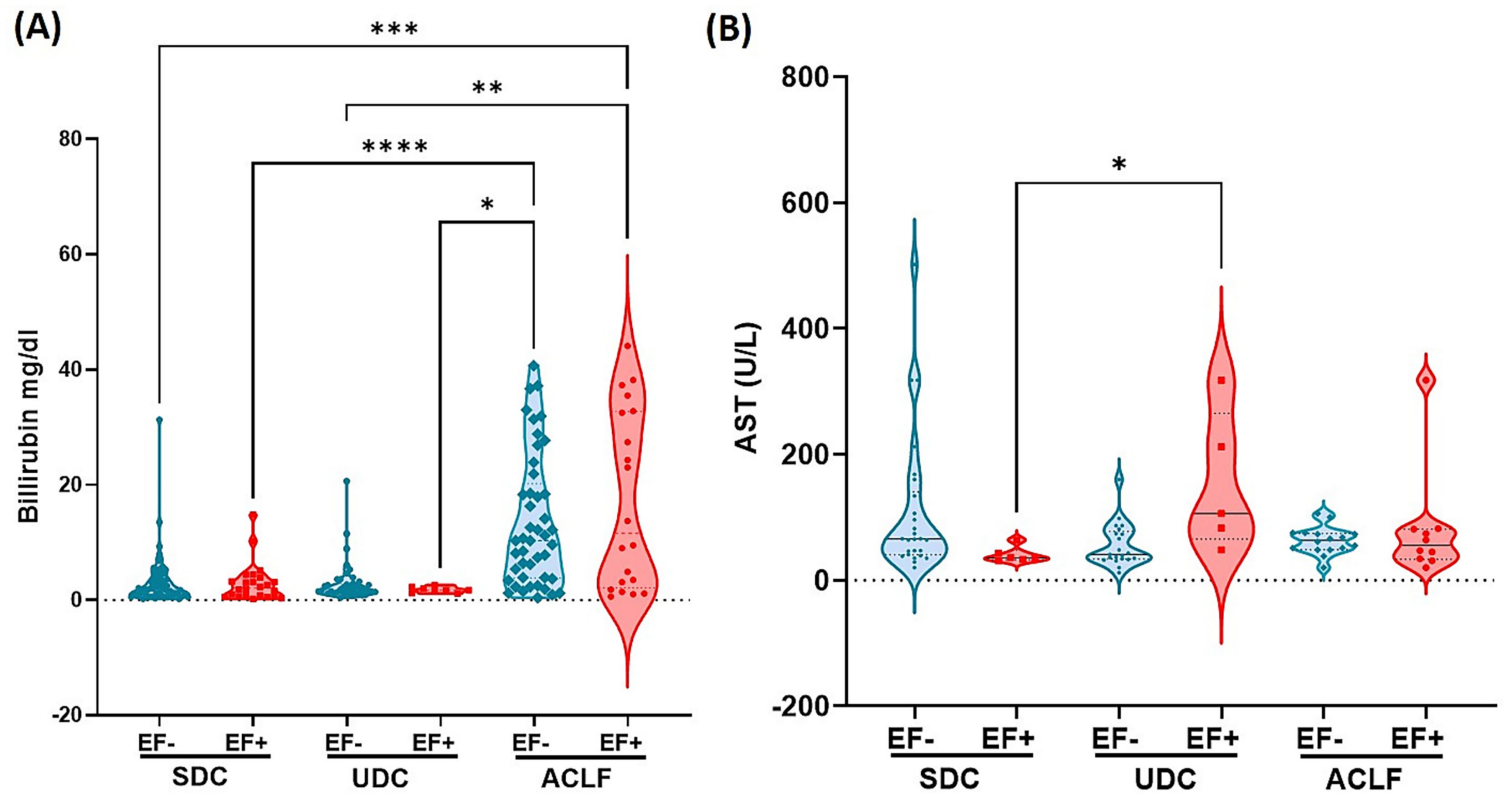
*E. faecium* DNA positivity is linked to altered liver function markers: elevated bilirubin and AST across cirrhosis stages. The clinical parameters associated with liver function include plasma levels of Bilirubin **(A)** and Aspartate Aminotransferase (AST) **(B)**. Significant differences between *E. faecium* (EF) DNA-negative (EF-) and EF DNA-positive (EF+) patients, as well as among the patient groups—stable decompensated cirrhosis (SDC), unstable decompensated cirrhosis (UDC), and acute-on-chronic liver failure (ACLF)—are indicated by asterisks (*p< 0.05, **p< 0.005, ***p< 0.0005, ****p< 0.0001). *p*-values were determined using one-way ANOVA with the Kruskal-Wallis test. Color codes: turquoise = EF-; red = EF+.

Our analysis of liver inflammation markers revealed significant variations in plasma bilirubin and AST levels across disease phenotypes and EF DNA status (Figure 3). Plasma bilirubin levels (Figure 3A) were markedly elevated in ACLF EF+ patients, who showed the highest concentrations across all groups. Statistically significant differences were observed between ACLF EF+ and other subgroups, including SDC EF+, SDC EF-, UDC EF+, and UDC EF- (*p*≤0.05 to *p* ≤ 0.005; Figure 3A). These findings suggest that bilirubin elevation is primarily driven by disease severity rather than EF DNA status. Serum AST levels (Figure 3B) were less different between the groups with UDC EF+ patients showing the highest levels which were significantly elevated compared to SDC EF+ patients (*p*≤ 0.05; Figure 3B). However, no significant differences were detected between EF+ and EF- patients within the same phenotype. The findings related to liver function emphasize the link between liver dysfunction markers and disease severity, particularly in ACLF patients. While EF DNA presence does not independently alter bilirubin or AST levels within each disease stage, it may contribute to worsening liver function in more advanced phenotypes such as UDC and ACLF.

### EF DNA positivity is associated with elevated creatinine levels and with worsened kidney function in ACLF patients

**Figure 4:**
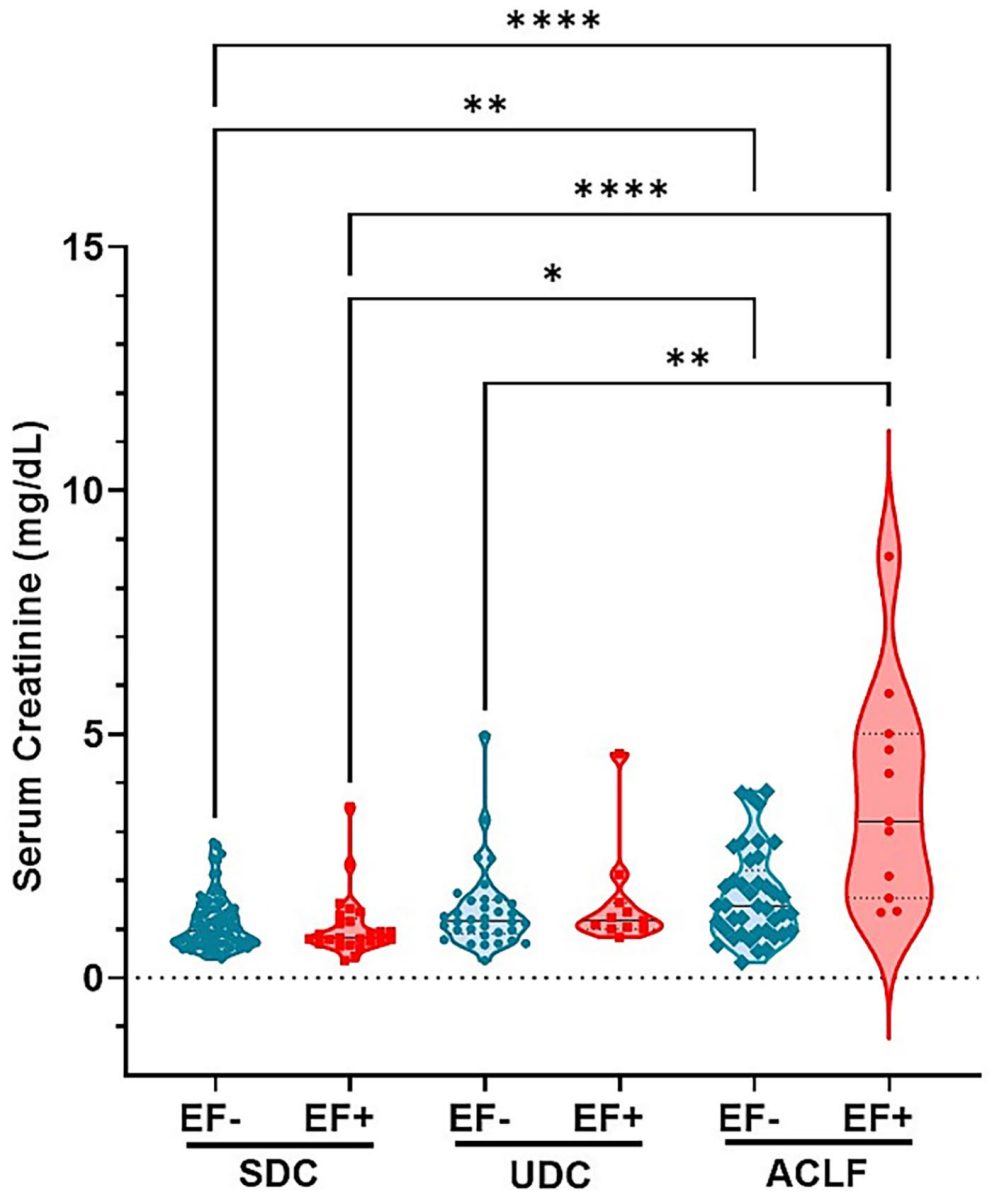
*E. faecium* DNA positivity is associated with elevated creatinine levels, indicating worsened kidney function across cirrhosis stages. Shown are serum levels of Creatinine. Significant differences between patients classified as *E. faecium* (EF) DNA negative (EF-) and EF DNA positive (EF+), as well as between the respective patient groups—stable decompensated cirrhosis (SDC), unstable decompensated cirrhosis (UDC), and ACLF (acute-on-chronic liver failure)—are indicated by asterisk (**p< 0.005; ****p< 0.0001). *p*-values were determined using one-way ANOVA with Kruskal-Wallis test. Color codes: turquoise = EF-; red = EF+.

Analysis of plasma creatinine levels revealed a correlation EF DNA detection in patient sera and kidney function (Figure 4). Creatinine concentrations were the highest in ACLF EF+ patients compared to most other subgroups including SDC EF-, SDC EF+ and UDC EF- groups, indicating more severe kidney dysfunction in this group (*p*≤ 0.0001 to *p* ≤ 0.005). Additionally, creatinine levels in ACLF EF- patients were significantly higher than those in SDC EF+ and SDC EF- groups (*p*≤ 0.005), further underscoring the impact of disease severity on renal function (Figure 4). Among the SDC and UDC groups, creatinine levels were relatively lower and showed no statistically significant differences between EF+ and EF- patients. These findings indicate that the presence of EF DNA is associated with worsened kidney function in patients with ACLF, while its impact is less evident in earlier stages of liver disease. Despite marked differences in creatinine levels, sodium and potassium concentrations remained consistent across all patient groups (data not shown). These results indicate that the presence of EF DNA is associated with more severe kidney dysfunction, particularly in ACLF patients, where SI and renal impairment are most pronounced.

### Association between EF DNA positivity and clinical parameters

We additionally examined how the presence of EF DNA relates to clinical data and its possible contribution to the development of ACLF. To do this, we used Random Forest and correlation analyses on a subset of 106 patients from our clinical dataset to assess how EF DNA affects different clinical parameters. Supplementary Figure 5A presents the top 25 clinical parameters most closely associated with EF DNA positivity within this cohort. Notably, serum levels of Macrophage Inhibitory Factor (MIF) emerged as the most significant clinical predictor of ACLF, showing a statistically significant correlation (*p* = 0.013). Additionally, while IL-18, platelet count, IL-1*β*, albumin, IFN-*γ*, IL-10, IL-1*α*, IL-6, bilirubin, serum creatinine, IL-1ra, INR, and MELD score were positively correlated with ACLF development, these associations did not reach statistical significance. In contrast, markers such as GGT, TNF-β, TNF-α, AST, ALT, hemoglobin, and hematocrit showed negative correlations with ACLF development, though these correlations were also non-significant. (Supplementary Figure 5A). We also examined which laboratory parameters were linked to the presence of EF DNA using the random forest classification, identifying Macrophage Inhibitory Factor (MIF), platelet count and sodium as most important predictors (Supplementary Figure 5B).

### Effect of EF DNA positivity on the cumulative stay in hospital

Cumulative hospital days were stratified by disease stage (SDC, UDC, ACLF) and *Enterococcus faecium* DNA status. Within each disease class (SDC, UDC, ACLF), no significant differences in cumulative hospital stay were observed between EF- and EF+ patients (Supplementary Figure 6A). However, a clear trend was noted in the ACLF group, where EF+ patients showed longer cumulative hospital stays compared to EF- cases. Overall, cumulative hospitalization was primarily determined by disease severity, with ACLF patients experiencing the longest stays regardless of EF status (Supplementary Figure 6A).

### Effect of EF DNA positivity on the length of hospital stay

The length of hospital stay was assessed across disease stages (SDC, UDC, ACLF) in relation to EF DNA status. No significant differences were found between EF- and EF+ patients within any disease class. In ACLF, EF+ cases showed a tendency toward longer hospital stays compared to EF- cases, although this trend did not reach statistical significance (Supplementary Figure 6B).

## Discussion

*Enterococcus faecium* is increasingly recognized as an important factor in liver disease and liver failure, primarily due to its role in infections that can worsen patient conditions. In patients with liver cirrhosis or ACLF, EF is found in infections such as spontaneous bacterial peritonitis and/or bloodstream infections, and has been linked to high rates of septic complications, which significantly can impact patient outcomes (35, 36). This study aimed to evaluate serum levels of *E. faecium* (EF) DNA in patients with liver disease, specifically investigating its association with systemic inflammation and organ dysfunction, as well as its potential as a biomarker for disease severity in decompensated cirrhosis and ACLF. A key finding was that EF DNA was detectable in approximately 26% of patients with decompensated liver cirrhosis, while it was almost absent in healthy individuals (around 1.3%), highlighting a potential link between EF DNA presence and advanced liver disease. These findings support the hypothesis that translocation of EF DNA into the bloodstream may contribute to SI and organ dysfunction in patients with decompensated cirrhosis and ACLF, potentially serving as an early marker of disease progression and severity. The characteristics of our patient cohort align closely with those of the CANONIC cohort (17), which originally defined the ACLF criteria using the CLIF-SOFA score to assess organ failure. Among ACLF patients, the distribution of EF DNA status was fairly similar, with 37% testing EF DNA-positive and 32% testing EF DNA-negative. However, inflammatory parameters such as leukocyte counts, IL-6, and CRP increased progressively with disease severity and were the highest in ACLF patients, particularly those who were EF DNA-positive (EF+), suggesting bacterial translocation and systemic inflammation (SI) as key factors in ACLF pathophysiology (10, 15, 36–38). The increased leukocyte count suggests an active immune response, likely triggered by bacterial translocation, while elevated bilirubin and AST indicate hepatic injury. The pro-inflammatory cytokine IL-6 stood out as the key cytokine, significantly elevated in EF+ ACLF patients, which reinforces its involvement in immune dysregulation and its potential as a disease marker (37, 39–43). This is in agreement with earlier studies linking IL-6 to SI, hepatic decompensation, and renal impairment in cirrhosis and ACLF patients (8, 20, 21, 42, 44, 45). Hepatic injury markers, like bilirubin followed a similar pattern, with significantly higher levels in EF+ ACLF patients, further confirming the association between disease severity and organ dysfunction (7, 45–48). Moreover, the presence of EF DNA was associated with significant increased portal hypertension in ACLF patients, indicating a compromised intestinal barrier integrity and enhanced microbial translocation (49–51). This aligns with current research noting that cirrhosis and portal hypertension promote bacterial translocation via a so called “leaky gut,” which in turn enhances SI (12, 50, 52, 53). Moreover, increased IL-6 levels are associated with SI, disease progression, and bacterial infections, all of which are known to worsen outcomes in patients with ACLF (45, 54, 55). However, and most interesting, our study identified a novel and robust association between EF DNA positivity and impaired kidney function in ACLF patients. Serum creatinine levels were elevated in EF+ ACLF patients compared to all other subgroups, suggesting dysfunction not explained solely by liver disease severity in ACLF patients (45, 46, 56). This finding points toward a potential role of EF in the development or exacerbation of kidney injury in ACLF, highlighting EF DNA as a potential indicator for kidney- dysfunction and intestinal barrier failure. While sodium and potassium levels remained stable across groups, the rise in creatinine in EF+ patients underscores kidney involvement as a critical and novel finding. In ACLF, renal dysfunction frequently presents as acute kidney injury (AKI) and may advance to hepatorenal syndrome (HRS), with inflammatory mechanisms playing a central role in this progression (13, 57, 58). (59, 60). Moreover, the cytokine storm characteristic of ACLF—dominated by elevated levels of IL-6 and TNF-*α*—leads to endothelial injury, glomerular damage, and diminished renal perfusion, compounding the deterioration of renal function (61–63).

The presence of EF and its association with the observed cytokine profiles suggest a link to progressive immune and organ dysfunction (42, 45, 49, 64). Our data therefore supports the use of IL-6, along with leukocyte count, CRP, bilirubin, and serum creatinine, as potential biomarkers to monitor disease progression and the inflammatory burden associated with EF DNA positivity in patients with decompensated cirrhosis and ACLF (36, 65–69).

To ensure the reliability of our RT-qPCR results and exclude the possibility of DNA contamination, stringent negative controls were implemented throughout the workflow. DNA extraction was performed under sterile conditions in a disinfected laminar flow cabinet using a validated chemical and UV-based decontamination protocol (28, 29). The detection of EF DNA in serum may have immunological relevance, as bacterial DNA acts as a potent pro-inflammatory stimulus. Unmethylated CpG motifs in bacterial genomes are recognized by Toll-like receptor 9 (TLR9), triggering innate immune responses and cytokine release, including TNF-*α* and IL-6 (70, 71). Although the pro-inflammatory potential of EF DNA has not been extensively studied, it likely exhibits similar effects, especially in the context of intestinal barrier dysfunction and bacterial translocation. Clinically, EF or its DNA in extraintestinal sites has been linked to systemic inflammation, immune activation, and adverse outcomes in patients with liver disease and critical illness (72, 73). These findings support the concept that translocated EF DNA may function as a microbial-associated molecular pattern (MAMP), contributing to systemic inflammation in acute decompensation and ACLF. Moreover, the markedly higher detection rate of EF DNA by RT-qPCR compared to conventional microbiological diagnostics, such as blood cultures and swab tests suggests that molecular approaches may serve as more sensitive indicator of clinically relevant bacterial translocation. The limited detection by culture-based methods likely reflects their focus on active infections or colonization, whereas RT-qPCR identifies a broader range of patients with translocated microbial DNA. This diagnostic discrepancy highlights the potential utility of serum-based molecular diagnostics for improving the detection of subclinical microbial translocation and its inflammatory consequences in liver disease.

Our findings show that renal failure in ACLF is not merely a consequence of hepatic decline but a parallel and immune-driven pathology, particularly intensified by EF DNA positivity. The inclusion of inflammatory markers such as IL-6 and neutrophil activation profiles alongside classical renal function markers like creatinine could provide earlier identification of high-risk patients and can help to guide to more targeted interventions (74–77).

## Data Availability

The original contributions presented in the study are included in the article/Supplementary material, further inquiries can be directed to the corresponding authors.

## Footnotes

^1^ www.graphpad.com
