# Supplementary material for: Enterococcus faecium DNA in acute decompensated cirrhosis: a key player in inflammation and kidney dysfunction
Source: Front Med (Lausanne). 2025 Oct 20;12:1629210. doi: 10.3389/fmed.2025.1629210 (PMC12580341; doi:10.3389/fmed.2025.1629210)
Supplement: Supplementary file 1 [file Data_Sheet_1.pdf]

Supplementary Material

Enterococcus faecium DNA in acute decompensated cirrhosis: a key player in inflammation and kidney dysfunction

Olaf Tyc[1], Nico Kraus[1], Toska Wiedemann[1], Hans-Peter Erasmus[1], Cristina Ortiz[1], Jessica Vasseur[2], Kai Hourfar[3], Christian Seidl[3], Marcus Maximilian Mücke[1], Holger Storf[2], Iulia Dahmer[4], Eva Herrmann[4], Stefan Zeuzem[1], Volkhard A. J. Kempf[5], Jonel Trebicka[1,6], Christoph Welsch[1] and Angela Brieger[1]

Affiliations:

[1] Goethe University Frankfurt, University Hospital, Medical Clinic 1, Biomedical Research Laboratory, Frankfurt am Main, Germany

[2] Goethe University Frankfurt, University Hospital, Institute of Medical Informatics, Frankfurt am Main, Germany

[3] Institute for Transfusion Medicine and Immunohematology, German Red Cross Blood Donation Service Baden-Wuerttemberg-Hessen, Frankfurt am Main, Germany

[4] Goethe University Frankfurt, University Hospital, Institute of Biostatistics and Mathematical Modeling, Frankfurt am Main, Germany

[5] Goethe University Frankfurt, University Hospital, Institute for Medical Microbiology and Infection Control, Frankfurt am Main, Germany

[6] Department of Internal Medicine B, Faculty of Medicine, University of Münster, Münster, Germany

Correspondence: Olaf Tyc (O.T.), Goethe University Frankfurt, University Hospital, Department of Internal Medicine I, Frankfurt, tyc@med.uni-frankfurt.de, Tel.: +49-69 6301-80464; a.brieger@em.uni-frankfurt.de (A.B.); Tel.: +49-69-6301-6218

Keywords: bacterial translocation, *Enterococcus faecium*, acute-on-chronic-liver-failure, chronic liver diseases, inflammation, gut barrier, gut dysbiosis, microbiome

Running Title: *Enterococcus faecium* DNA in ACLF patients

Supplementary Tables

Supplementary Table 1: All bacterial strains used during this study.

Strain / isolate / organism	Phylum/class	Function
Enterococcus faecium (DSM# 20477)	*Bacilli*	DNS isolation and cloning
Escherichia coli Dh5α	*Gammaproteobacteria*	Cloning, DNA isolation for qPCR standards
Escherichia coli JM109	*Gammaproteobacteria*	Cloning, DNA isolation for qPCR standards

Supplementary Table 2: Primers used in this study.

Primers used in this study

Primer code	Sequence (5' - 3') of primers	target	product size	reference
E._faecium_qPCR_16SF	GCGGCTCTCTGGTCTGTAAC	16S rDNA gene *E. faecium*		this study
E._faecium_qPCR_16SR	TAAGGTTCTTCGCGTTGCTT	16S rDNA gene *E. faecium*	254 bp	this study

Supplementary Figures

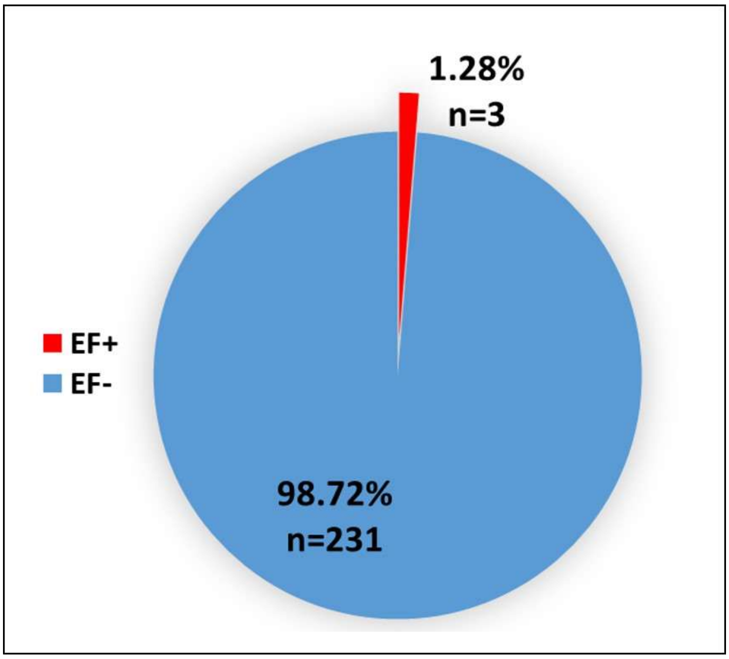

Supplementary Figure 1: RT-qPCR Results of the healthy control cohort. Analysis of serum samples from healthy individuals in the control cohort, categorized as *Enterococcus faecium* (EF) DNA-positive (EF+) or EF DNA-negative (EF-).

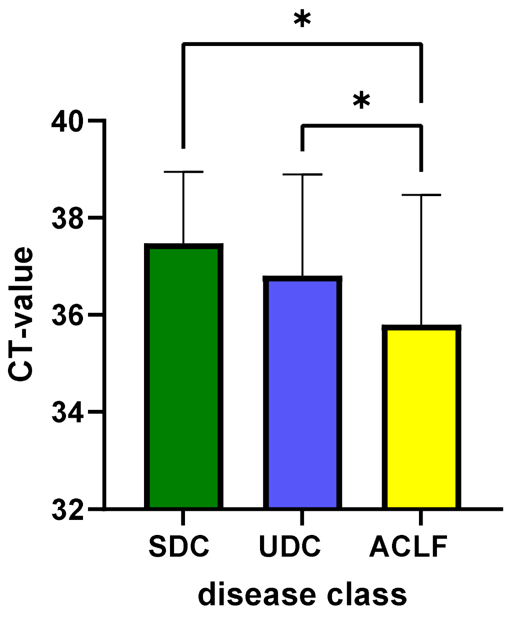

Supplementary Figure 2: **RT-qPCR Analysis of 16S rDNA Gene Reveals Lower Mean CT Values in ACLF Patients Compared to SDC and UDC**. The results of the RT-qPCR analysis of DNA samples from ACLF, SDC, and UDC patients showed significantly lower mean CT values in ACLF patient samples compared to those from SDC patients ($p<0.0001$) and UDC patients ($p=0.0254$). P-values were calculated using one-way ANOVA with the Kruskal-Wallis test.

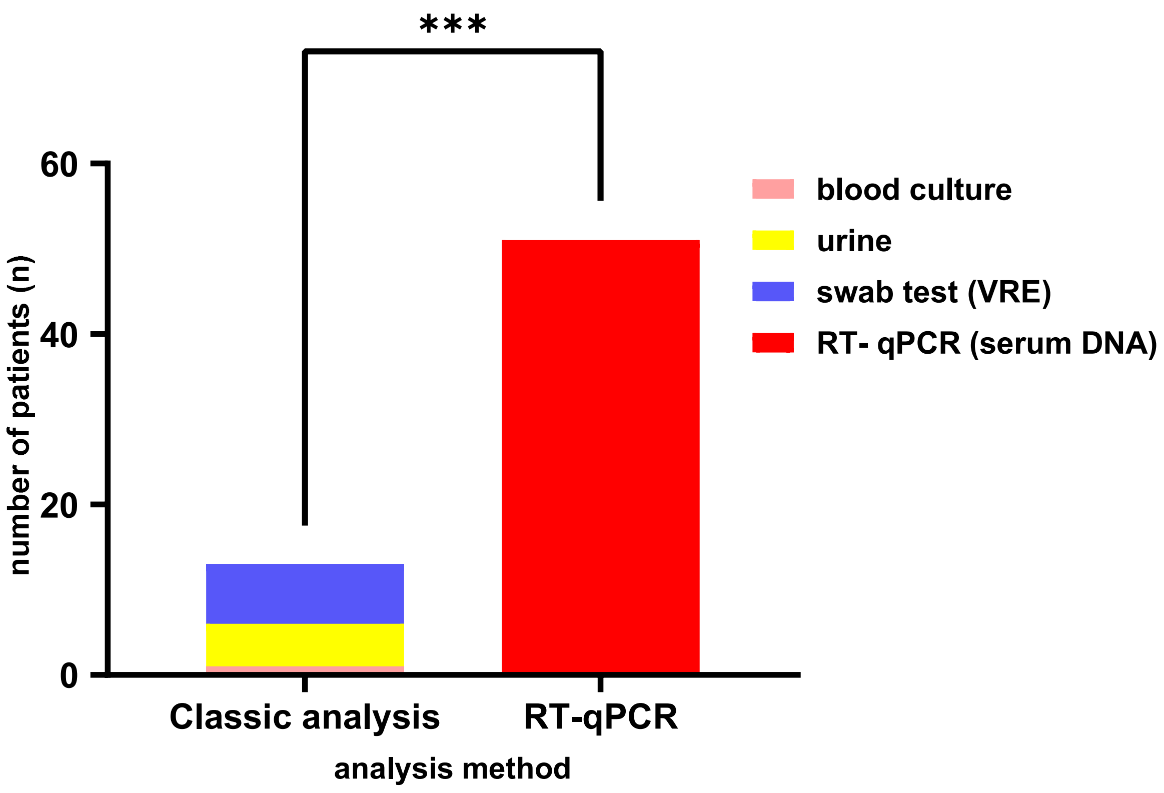

Supplementary Figure 3: Enhanced Detection of EF-Positive Patients Using RT-qPCR Compared to Classical Microbiological Methods. Comparative analysis of EF-positive patient identification using classical microbiological methods (blood culture, urine, and VRE swab tests; total n=13) versus RT-qPCR analysis of serum DNA (n = 51). The RT-qPCR method identified a significantly higher number of EF-positive cases. A Chi-square test confirmed the statistical significance of this difference (***p < 0.0001), highlighting the superior sensitivity of RT-qPCR over traditional culture-based diagnostics in this patient cohort.

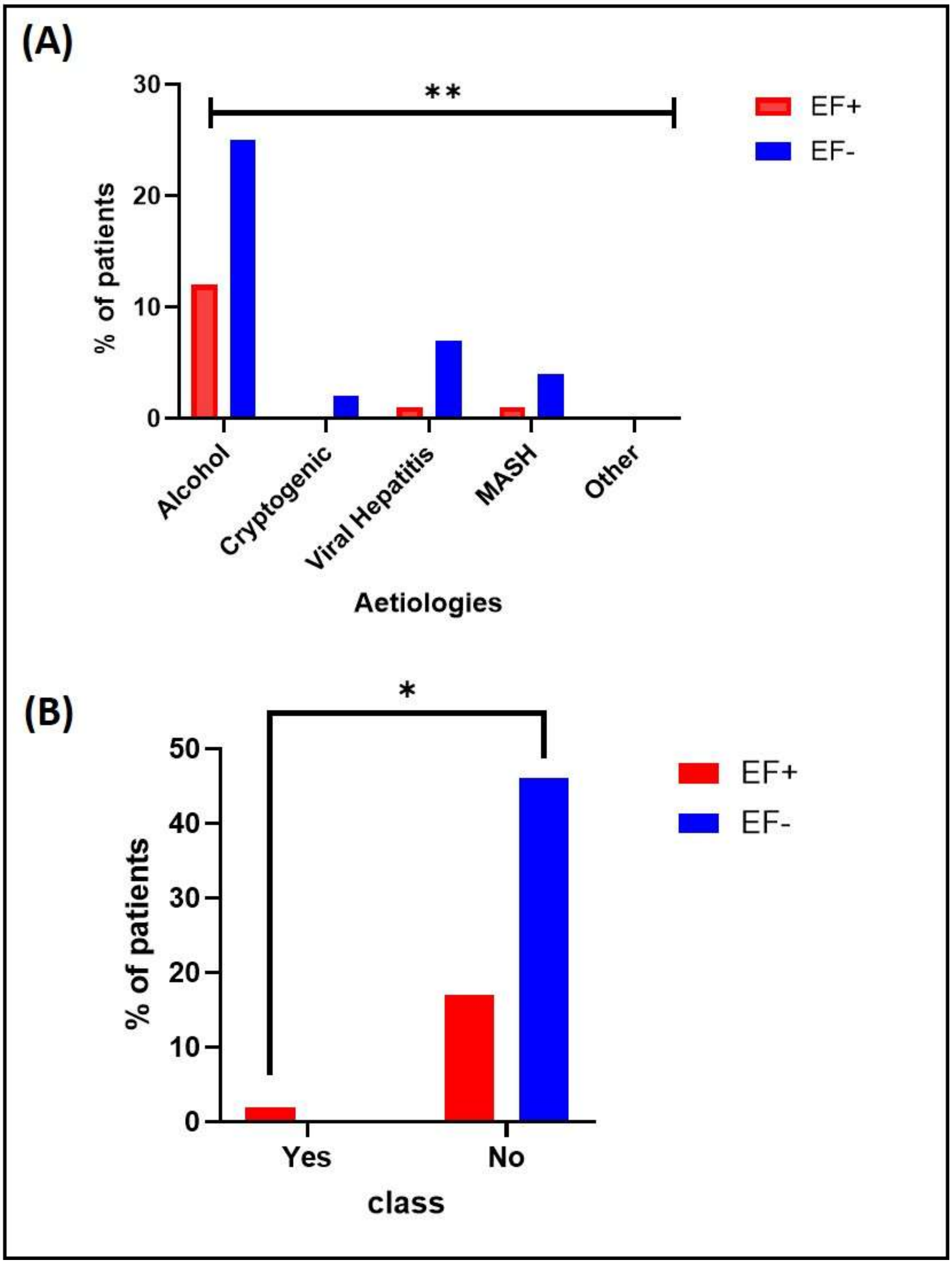

Supplementary Figure 4: **Differences in Disease Aetiology and Portal Hypertension between ACLF EF+ and EF− Patients.** The figure shows the statistical comparison of disease aetiologies—including alcohol-related, cryptogenic, viral hepatitis, MASH (Metabolic dysfunction-associated steatohepatitis), and other causes as well as the prevalence of portal hypertension in patients with ACLF, stratified by EF DNA status. A significant difference in aetiology distribution was observed between EF+ and EF− ACLF patients (Chi-square test, p = 0.008) (**A**). Additionally, portal hypertension was significantly more prevalent in one group, with a notable difference between EF+ and EF- ACLF patients (Chi-square test, p = 0.0254) (**B**). Colour code: Blue = EF-; Red = EF+.

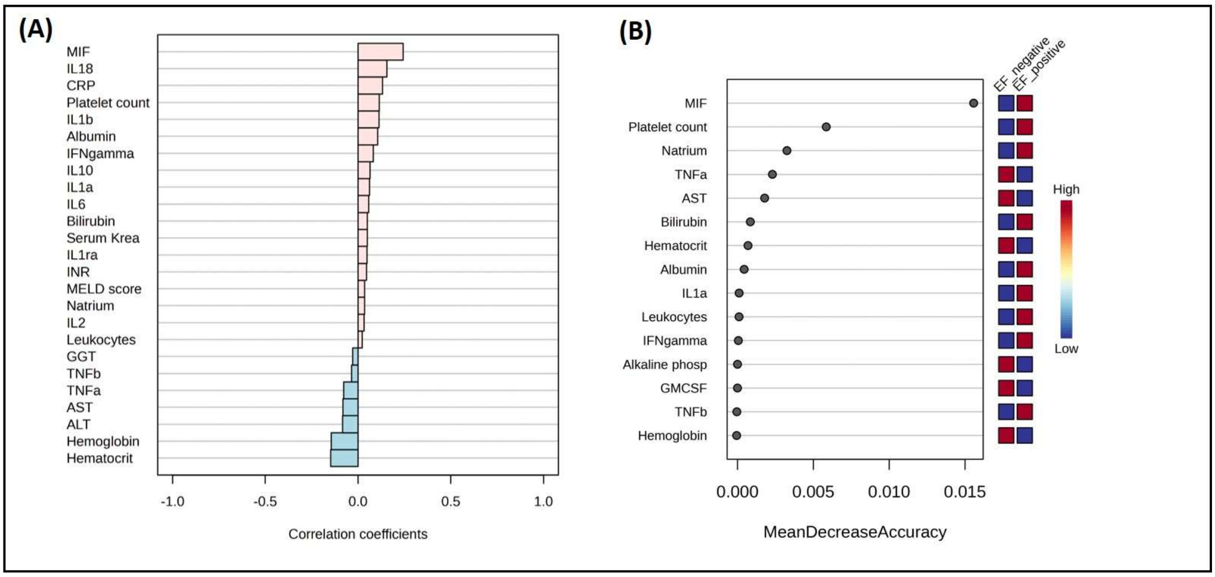

Supplementary Figure 5: **Metadata and Correlation Analysis of Clinical Data Investigating the Relationship between *Enterococcus faecium* DNA and ACLF**. A comprehensive correlation analysis was performed on the clinical data to investigate the relationship between the presence of EF DNA and ACLF. The top 25 clinical parameters associated with EF DNA presence were analyzed using Kendall's correlation, identifying serum potassium levels as a strong predictor (**A**). Laboratory parameters associated with the presence or absence of EF DNA were assessed, with Macrophage Migration Inhibitory Factor (MIF) emerging as the most significant laboratory parameter (**B**).

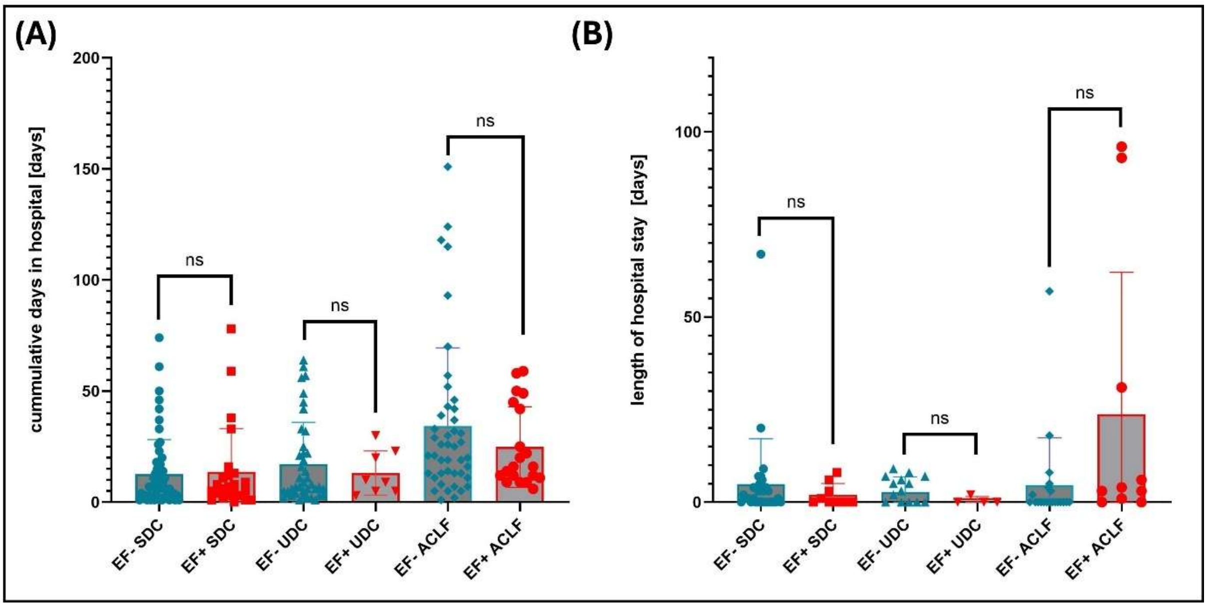

Supplementary Figure 6 Effect of *E. faecium* DNA positivity on the cumulative days in hospital and the length of hospital stay. (A) Cumulative hospital days stratified by disease stage and EF DNA status. Patients were grouped into stable decompensated cirrhosis (SDC), unstable decompensated cirrhosis (UDC), and acute-on-chronic liver failure (ACLF), and further stratified by EF- (turquoise) and EF+ (red). Within each disease class, no significant

differences were detected between EF– and EF+ patients (ns). P-values were determined using one- way ANOVA with the Kruskal- Wallis test. **(B)** Length of hospital stay stratified by disease stage and EF DNA status. Patients were grouped into SDC, UDC, and ACLF, and further separated by EF- (turquoise) and EF+ (red). No significant differences were detected within each disease class (ns). P-values were determined using one- way ANOVA with the Kruskal-Wallis test.
